# Isoschaftoside Reverses Nonalcoholic Fatty Liver Disease via Activating Autophagy *In Vivo* and *In Vitro*

**DOI:** 10.1155/2022/2122563

**Published:** 2022-06-27

**Authors:** Yanze Su, Yixing Kang, Jing Yi, Qirui Lin, Chaochuang Zhang, Zewei Lin, Zilong Yan, Jianhua Qu, Jikui Liu

**Affiliations:** ^1^Department of Clinical Medicine, Weifang Medical University, Weifang 261031, China; ^2^Department of Hepatobiliary and Pancreatic Surgery, Peking University Shenzhen Hospital, Shenzhen 518036, China

## Abstract

Nonalcoholic fatty liver disease (NAFLD) is the most common metabolic liver disease globally, and the incidence of NAFLD has been increasing rapidly year by year. Currently, there is no effective pharmacotherapy for NAFLD. Therefore, studies are urgently needed to explore therapeutic drugs for NAFLD. In this study, we show that isoschaftoside (ISO) dramatically reduces lipid deposition in cells. Meanwhile, ISO treatment reverses the NAFLD and reduces hepatic steatosis in mice. Importantly, we reveal that ISO suppresses the expression of light-chain 3-II (LC3-II) and SQSTM1/p62 in palmitic acid (PA) induced autophagy inhibition in the cell model and the NAFLD mouse model, which suggests that ISO might reverse NAFLD through regulating autophagy flux. We propose that ISO might alleviate hepatic steatosis in NAFLD via regulating autophagy machinery. Consequently, our study suggests that ISO might be of potential clinical value in the field of NAFLD therapy. ISO might have the potential for future therapeutic application.

## 1. Introduction

NAFLD is a disease characterized by liver parenchymal cell steatosis and intracellular lipid storage [1], which is usually connected with insulin resistance, obesity, diabetes, and other diseases [2]. NAFLD also affects the metabolism of substances in the body, which can bring about a range of changes in blood lipid and blood sugar levels [3, 4]. The morbidity of NAFLD has been rising year by year in both developed and developing countries [5]. The prevalence of NAFLD is increasing in all populations. More seriously, NAFLD can develop into chronic hepatitis, cirrhosis, and finally liver cancer [1]. Although lots of studies have been done on NAFLD, the pathogenesis of NAFLD is too complex to be fully elucidated. To date, there is still a lack of accurate and efficient curative drugs for NAFLD treatment in clinics. Therefore, finding an effective treatment for NAFLD is a good choice.

It was proposed that lipid metabolism disorders lead to lipid accumulation in liver cells [6]. Additionally, autophagy is a crucial mechanism to regulate lipid metabolism [7]. The autophagy-regulating lipid metabolism in the liver is called lipophagy, and lipids can be decomposed into free fatty acids through lipophagy, which can be reused by the body [8]. It has been reported that the autophagy in NAFLD could be inhibited in the presence of a high-fat diet or inducers, which results in lipid dysfunction and impaired autophagy flux, leading to lipid deposition in the liver [9].

Recently, many studies have demonstrated that the extracts of Chinese herbal medicine could activate autophagy to improve the severity of fatty liver and reduce steatosis and liver inflammation [10, 11]. The application of Chinese herbal medicine promises to be an important means of treating NAFLD. However, its specific mechanism of action in the field still needs further investigation. Studies indicated that the therapeutic treatment of NAFLD by Chinese herbal medicine has attracted more and more attention [12]. Chinese herbal medicine is a kind of natural ingredient extracted from plants. Compared with Western medicine, it has relatively lower rates of toxicity and side effects. Studies have shown that effective ingredients extracted from Chinese herbal medicine have been demonstrated to play important roles in the clinical effectiveness of NAFLD treatment. Clinical studies proposed that Chinese herbal medicine achieved a good clinical therapeutic effect in NAFLD treatment [13]. To date, the Chinese herbal extracts were shown to relieve NAFLD in numerous aspects, such as lipid metabolism abnormalities, endoplasmic reticulum stress, oxidative stress, inflammation, hepatocyte apoptosis, and necrosis [3, 4]. Silybin and berberine have been used in phase 4 clinical studies in the United States [14]. Silybin can reverse NAFLD by reducing oxidative stress and inflammatory response [15]. Berberine has a certain effect on endoplasmic reticulum stress and inflammation in NAFLD [16]. Recent studies have shown that extracts of Chinese herbal medicine can induce autophagy regulation, which is one of the relevant mechanisms involved in NAFLD. Therefore, Chinese herbal medicine-induced autophagy activation is a direction for NAFLD therapeutic treatment.

Isoschaftoside (ISO, C26H28O14; Supplementary Figure 1(a)) is a Chinese herbal monomer extracted from Abrus cantoniensis Hance, one of the flavonoids' active components [17]. Flavonoids are a kind of Chinese herbal medicine for the treatment of NAFLD. The active components of total flavonoid glycoside C include vicenin-2, isoschaftoside, and schaftoside [18]. Studies have shown that vicenin-2 could regulate inflammatory pathways in the treatment of NAFLD [19]. The studies published in the Chinese Journal of Traditional Chinese Medicine (2017, 32 (11):5078–5081) proposed the protective effect of schaftoside on NAFLD induced by a high-fat diet in mice. The vicenin-2 and schaftoside have been shown to have therapeutic effects on NAFLD. Therefore, we speculated that isoschaftoside might have therapeutic effects on NAFLD. Studies have shown that in traditional Chinese medicine Abrus cantoniensis Hance has the function of soothing the liver and promoting gallbladder and can be used to treat hepatitis [20, 21], but the specific effective composition is not clear. To date, the research on the effects of ISO is still at an early stage. In this study, we demonstrated that ISO could reverse hepatic steatosis in NAFLD through regulating autophagy flux.

## 2. Material and Methods

### 2.1. Chemicals, Reagents, and Antibodies

The chemicals and reagents were from the following sources: ISO (Chengdu Must Bio-Technology Co., Ltd, 52012-29-0), sodium palmitate (Sigma, P9767-5G) that was used to make PA, Dulbecco's modified Eagle's Medium (DMEM, Corning, 10013072), bovine serum albumin (BSA, FDbio Science, FD0030), pentobarbital sodium (Sigma, CAS: 57-33-0), CCK-8 Kit (Beyotime, C0039), Caspase-3 Activity Assay Kit (Solarbio, BC3830), DAPI (Beyotime, P0131-25 ml), Oil Red O Staining Kit (Beyotime, C0157), and chloroquine (CQ, MCE, HY-17598A). The antibodies that we used in the study were from the following sources: LC3B Rabbit mAb (ABclonal, A19665), SQSTM1/p62 Rabbit mAb (ABclonal, A19700), ATG5 Rabbit mAb (ABclonal, A19677), Beclin-1 Rabbit mAb (ABclonal, A7353), anti-mTOR antibody (Abcam, ab32028), phospho-mTOR-S2448 Rabbit mAb (ABclonal, AP0115), *β*-actin monoclonal antibody (ProteinTech, 66009-1-l g), Goat Anti-Mouse IgG H&L (HRP) (Abcam, ab6789), Goat Anti-Rabbit IgG H&L (HRP) (Abcam, ab6721), and Goat Anti-Rabbit IgG H&L (Alexa Fluor 488, ab150077).

### 2.2. Cell Culture and Treatment

HepG2 cells were purchased from Procell Life Science & Technology Co., Ltd. The cells were cultured in DMEM high-glucose medium containing 10% fetal bovine serum (FBS) and 1% penicillin/streptomycin (Gibco, USA) in a 37°C 5% CO_2_ incubator. The preparation of 10 mM PA is referred to the existing literature [22]. Sodium palmitate in PBS was incubated at 70°C for 30 minutes and mixed with 30% BSA in PBS at a 1 : 1 volume ratio of PA/BSA. 0.4 mM PA was used to induce autophagy cell models. 15% BSA was used as a blank control. We labeled blank control as the NC group. To establish the cell model of NAFLD *in vitro*, PA was added into the medium at a concentration of 0.4 mM. In *in vitro* experiment, we used different concentrations of ISO and 0.4 mM PA or equivalent BSA solution to culture cells for 24 h.

### 2.3. CCK-8 Assay

We seeded 5 × 10^3^ HepG2 cells on 96-well plates. After 24 h, the medium was replaced with fresh medium with various concentrations of PA. Following 24 h of treatment, we added 10 *μ*l CCK-8 solution and 100 *μ*l fresh medium into each well. The absorbance was measured by spectrophotometer at 450 nm wavelength after 2 h of treatment.

### 2.4. Oil Red O Staining to Evaluate the Level of Lipid Deposition

Oil Red O Staining of Cells: after washing the cells twice with PBS, we fixed the cells with 4% paraformaldehyde for 10–15 minutes and stained the cells with the Oil Red O Kit; Oil Red O Staining of Liver Tissues: liver tissues were immediately frozen in liquid nitrogen after harvesting. Then, the samples were embedded. The frozen liver slices were prepared and stained with Oil Red O Staining Kit. Three random images were captured using a light microscope (Olympus BX53) for each sample.

### 2.5. Western Blot

The total protein was extracted with RIPA buffer (containing 0.1% SDS, 1% protease inhibitor, and 1% phosphatase inhibitor; Solarbio, R0020). Protein quantification was performed using the BCA Kit (Coolaber, SK1070-5000T). 20 *μ*g protein lysis was prepared for SDS-PAGE (6% concentrated gel and 12% and 15% separated gel) and transferred to 0.22 *μ*m Immobilon-PSQ PVDF membrane (Merck Millipore Ltd., ISEQ00010). The membranes were blocked with 5% nonfat-dried milk (Coolaber, CN7861-500G) at room temperature for 2 h, washed 3 times with TBST (Solarbio, No. T1080) for 15 minutes each time, and hatched with primary antibody at 4°C overnight. After being washed, the membranes were hatched with the corresponding secondary antibody at room temperature for 1 h and then washed 3 times with TBST for 15 minutes each time. *β*-actin was used as an internal control. All immunoreactive protein bands were visualized by BeyoECL Moon (Beyotime, P0018FM-2) and quantified by software Image-Pro Plus 6.0.

### 2.6. Immunofluorescence for LC3B in Cells

1 × 10^5^ cells were plated in 24-well plates. After 24 h of PA and ISO treatment, the cells were fixed with 4% paraformaldehyde for 15 minutes, washed with PBS 3 times, and sealed with 5% BSA solution for 30 minutes, and 200 *μ*l LC3B (1 : 200) solution was added to the 24-well plate 4°C overnight and washed with PBS 3 times. Goat Anti-Rabbit IgG H&L (Alexa Fluor 488) solution (1 : 500) was added and incubated for 1 h, washed with PBS 3 times, sealed with DAPI, and observed under the fluorescence microscope.

### 2.7. Animal Experiments

We conducted NAFLD animal model using C57 mice fed with a high-fat diet [23, 24]. 20 C57 mice (4 weeks; male; C57BL/6JGpt) were purchased from Jiangsu Jicuiyaokang Biotechnology Co, Ltd., and all mice were kept in a pathogen-free facility at a specific temperature (24°C ± 5°C), a specific humidity (55% ± 5%), and a specific light (12 h of light/12 h of dark cycle). After 1 week of adaptive feeding, they were randomly divided into two groups. The control group (*n* = 6) was fed with an ordinary diet, and the high-fat diet group (*n* = 14) was fed with a high-fat diet, and their weight was recorded weekly for 16 weeks. Starting from the 17th week, the high-fat diet group was randomly divided into the HFD group (*n* = 7) and the ISO-treated group (*n* = 7). We considered the HFD group as the model group and continued to feed the HFD group and the ISO-treated group with a high-fat diet for 4 weeks. The ISO-treated group was given ISO (20 mg/kg/day) by intraperitoneal injection, and the control group and the HFD group were given equal 0.9% normal saline for 4 weeks. The body weight of mice was recorded weekly. All animal experiments were carried out in accordance with the Experimental Animal Welfare Act and approved by the Experimental Animal Welfare Ethics Committee. Our laboratory has a laboratory animal license.

### 2.8. Blood Biochemical Test

Blood samples were collected from each mouse by eyeball sampling. The serum was centrifuged at 3000 rpm for 10 minutes at 4°C and stored at −80°C. The serum levels of alanine transaminase (ALT), aspartate transaminase (AST), fasting blood glucose (GLU), total cholesterol (TC), and triglyceride (TG) were measured in Wuhan Xavier Biotechnology Co., Ltd., and the kits were purchased from Changchun Huili Biotechnology Co., Ltd.

### 2.9. Hematoxylin and Eosin (H&E) Staining

All fresh livers were washed with normal saline and fixed in a 10% neutral paraformaldehyde buffer solution. Paraffin-embedded liver biopsy sections (5 mm) were stained with H&E for histological analysis. The sections were observed and taken pictures with a light microscope (Olympus BX53) for changes in the organization structure.

### 2.10. Statistical Analysis

All data were represented by the mean ± standard deviation (SD) in three independent trials. The Mann–Whitney *U* test (nonnormally distributed variables) and t test (normally distributed variables) were used for comparison between two groups; the Kruskal–Wallis *H* test (nonnormally distributed variables) and one-way analysis of variance were used for comparison between multiple groups. Data were analyzed by GraphPad Prism 7.0 software. All data were represented by the mean ± standard deviation (SD) in three independent trials. ^*∗*^*P* < 0.05, ^*∗∗*^*P* < 0.01, ^*∗∗∗*^*P* < 0.001, and ^*∗∗∗∗*^*P* < 0.0001.

## 3. Results

### 3.1. ISO Reduces Lipid Deposition in Cells via Activating Autophagy Flux

**Figure 1:**
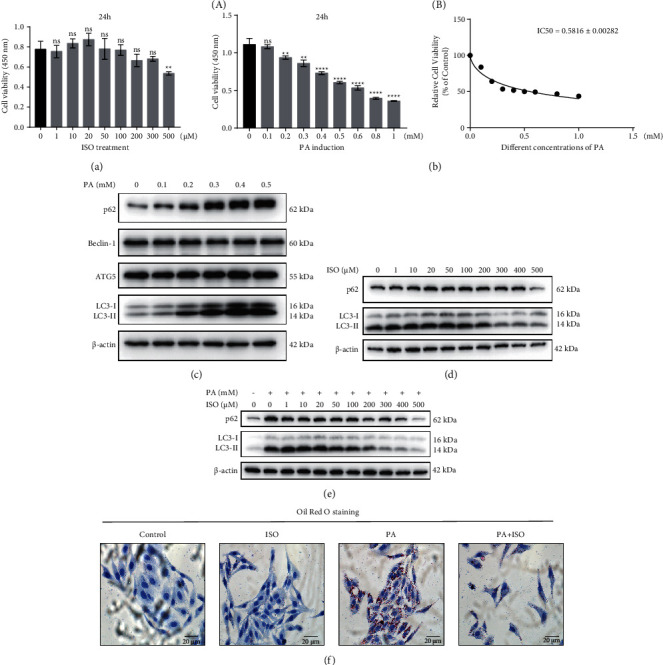
ISO reduces lipid deposition via activating autophagy flux *in vitro*. (a) Cell viability after different concentrations of ISO on HepG2 cells after 24 h. (b) Cell viability after different concentrations of PA on HepG2 cells after 24 h (ii). IC50 of PA (IC50 means half-inhibitory concentration). (c) The effects of different concentrations of PA on autophagy-related proteins. (d) The effects of different concentrations of ISO on p62 and LC3-II. (e) The effect of different concentrations of ISO on p62 and LC3-II in the presence or absence of 0.4 mM PA. (f) The cells were stained with Oil Red O Kit and photographed to observe intracellular lipid deposition (red staining). Scale bar: 20 *μ*m.

To investigate the therapeutic effect of ISO in NAFLD, we employed the cell model to evaluate the regulatory capacity of ISO in lipid deposition. The cytotoxicity assessments of ISO and PA were performed using a CCK-8 assay. The HepG2 cells were treated with a concentration gradient of ISO or PA. We observed that ISO had no significant impact on cell proliferation at a concentration below 500 *μ*M (Figure 1(a)). We used the Caspase-3 Activity Assay Kit to examine the variation of apoptosis of cells after ISO treatment. We found that ISO treatment did not affect cells significantly (Supplementary Figure 2(b)). As reported previously, 0.4 mM PA was used to repress autophagy in HepG2 cells [25, 26]. We also found that PA exhibited a dose-dependent induction of cytotoxicity in HepG2 cells (Figure 1(b) i), IC50 = 0.5861 ± 0.00282 mM (Figure 1(b) ii). To examine the PA-induced inhibition of autophagy flux, we examined p62, LC3-II, beclin-1, and ATG5 protein expression level using Western blot. LC3-II and p62 were the proteins related to autophagy substrates, and the increased expression of them indicated that the function of autophagy was inhibited. The expression levels of p62 and LC3-II increased as the concentration of PA increased, while no significant changes in the expression levels of beclin-1 and ATG5 were observed after PA treatment, and we used *β*-actin as an internal reference for quantitative analysis of autophagy-related protein (Figure 1(c)). Quantification of Western blot was performed using ImageJ (Supplementary Figure 1(b)). Therefore, we speculated that PA might block the fusion process of autophagosome and lysosome, leading to the accumulation of autophagic substrate proteins LC3-II and p62, while PA had no significant effect on the formation of autophagosome [27]. We suggested that PA might inhibit the downstream pathway of autophagy resulting in lipid deposition. The results revealed that we successfully established the PA-induced autophagy inhibition model in HepG2 cells in our study. Meanwhile, 0.4 mM of PA treatment was determined to be used for PA-induced autophagy inhibition model, which significantly inhibited autophagy with a relative lower cytotoxicity in HepG2 cells. We then treated cells with a concentration gradient of ISO and examined the effect of ISO treatment on autophagy in HepG2 cells. Autophagy flux was detected by Western blotting using LC3-II and p62 antibodies (Figure 1(d)). When the concentration of ISO treatment was higher than 200 *μ*M, we observed significant reductions in LC3-II expression. The results suggested that the autophagy was activated when the concentration of ISO treatment was increased to 200 *μ*M. We then examined the effect of ISO treatment on PA-induced autophagy inhibition in HepG2 cells. As shown in Figure 1(e), ISO significantly activated autophagy flux and eventually decreased LC3-II and p62 expression in PA-induced autophagy inhibition in HepG2 cells. Furthermore, the Oil Red O staining showed that the PA treatment dramatically increased the amount of intracellular lipid droplets compared with the control group (Figure 1(f), control group *vs*. PA group). The results revealed that we successfully constructed the PA-induced lipid deposition model in cells. We then examined the effect of ISO on lipid deposition accompanied by PA-induced autophagy inhibition. Intriguingly, ISO treatment decreased the intracellular lipid droplets in PA-induced lipid deposition model suggesting that ISO treatment alleviated lipid deposition in cells (Figure 1(f), PA group *vs*. PA + ISO group). Taken together, we revealed that ISO might suppress lipid deposition in PA-induced autophagy inhibition model via activation of autophagy flux.

### 3.2. Experimental Evidence for Autophagy Activation by ISO

**Figure 2:**
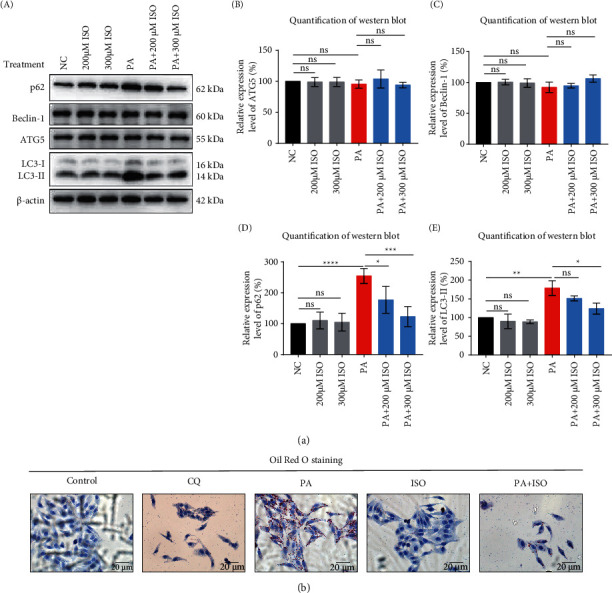
Experimental evidence for autophagy activation by ISO *in vitro*. (a) (i–vi). Western blot was performed to verify the effect of ISO and 0.4 mM PA on autophagy-related proteins and quantitative analysis. (b) In the pictures of HepG2 cells stained by Oil Red O Kit, compared with the control group, lipid deposition (red staining) was clearly observed in the PA group, and ISO could reduce PA-induced lipid deposition. CQ was used as the positive control for inhibition of autophagy, and the cells treated by 10 *μ*M CQ had lipid deposition. Scale bar: 20 *μ*m.

To further investigate the mechanisms of ISO-reduced lipid deposition via activating autophagy, we measured the expression of autophagy-related proteins such as LC3-II, p62, beclin-1, ATG5, mTOR, and p-mTOR in cells after different treatments. We observed that the expressions of LC3-II and p62 increased after PA treatment, and the increased expression of LC3-II was synchronized with the accumulation of p62, indicating that the autophagy flux was impaired (Figure 2(a) i, iv, v). Meanwhile, expression levels of LC3-II and p62 were reduced after ISO treatments at 200 *μ*M and 300 *μ*M (Figure 2(a) i, iv, v), indicating that ISO could restore the PA-induced reduction of autophagy flux. PA and ISO treatments had no significant effect on the expression levels of beclin-1, ATG5, mTOR, and p-mTOR, possibly because PA and ISO had no significant impact on the formation of autophagosome and the upstream of the autophagy pathway (Figure 2(a) and Supplementary Figure 1(c) i, ii, iii). The results of immunofluorescence showed that compared with the control group, there was an obvious green fluorescence accumulation in the PA group (Supplementary Figure 2(a), marked in red, control group *vs* PA group), indicating that PA-induced intracellular LC3B protein accumulation; compared with the PA group, the green fluorescence accumulation in the PA + ISO group was significantly reduced (Supplementary Figure 2(a), PA group *vs* PA + ISO group), indicating that ISO could reduce the LC3B protein accumulation induced by PA. We did not see green fluorescence in the ISO group compared with the control group (Supplementary Figure 2(a), control group *vs*. ISO group). The results suggested that ISO treatment dramatically downregulated the accumulation of LC3B in cells. Under the same experimental conditions, we further verified the association between autophagy and lipid deposition by inhibiting autophagy through CQ [28]. CQ is an inhibitor of autophagy, which can block the fusion of autophagosome and lysosome, thereby inhibiting the autophagy flux [28, 29]. We cultured HepG2 cells separately with CQ and then performed Oil Red O staining and Western blot to examine lipid deposition and autophagy flux. In the Oil Red O staining, compared with the control group, lipid deposition was significantly increased in the CQ group, indicating that CQ inhibited the autophagy flux of HepG2 cells, leading to lipid deposition (Figure 2(b), control group *vs* CQ group). In general, the inhibition of autophagy flux can lead to lipid deposition [30, 31], and the activation of autophagy flux can reduce lipid deposition, which also provides a theoretical basis for our experiment.

### 3.3. ISO Treatment Decreased Liver Steatosis in High-Fat Diet Mice

**Figure 3:**
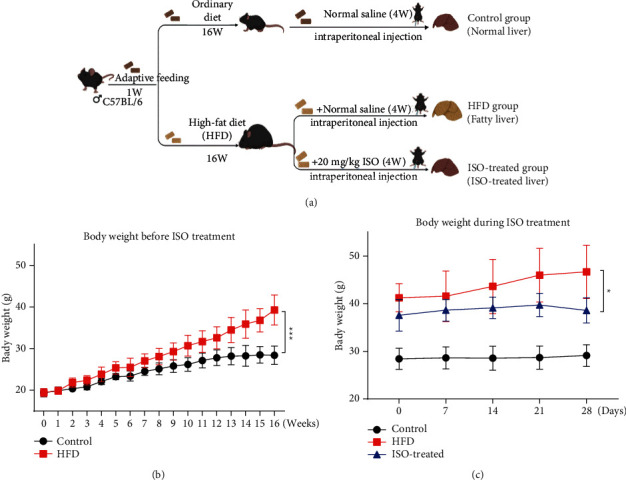
Animal experiment. (a) Schematic design of the experiment in mice. The control group: ordinary diet and intraperitoneal injection of normal saline. The HFD group: high-fat diet and intraperitoneal injection of normal saline. The ISO-treated group: high-fat diet and intraperitoneal injection of ISO (20 mg/kg/day). (b) Body weight before ISO treatment. (c) Body weight during ISO treatment.

To investigate the effects of ISO on high-fat diet mice, C57BL/6 mice were fed a standard purified rodent diet or a high-fat diet, respectively, for 16 weeks and then given ISO (20 mg/kg/day) or equivalent normal saline for 4 weeks (Figure 3(a)). From the first week to the 16th week, we recorded the changes in the body weight of the mice and conducted the statistical analysis. The experimental results showed that the body weight of mice in the HFD group was significantly higher than that in the control group, suggesting that the high-fat diet caused obesity in mice compared with the control group (Figure 3(b)). Based on the weight changes in the control group and the high-fat diet group, we believed that the establishment of the NAFLD animal model was successful. To explore the effect of ISO intervention on high-fat diet mice, we randomly divided the high-fat diet mice into two groups, and we got the HFD group and the ISO-treated group. The HFD group was the model group, and the ISO-treated group was fed with a high-fat diet and treated with ISO. The ISO-treated group was given ISO (20 mg/kg) by intraperitoneal injection once per day, and the control group and the HFD group were given equal 0.9% normal saline for 4 weeks, and the changes in body weight are shown in Figure 3(c). We could see that the body weight of the ISO-treated group was significantly lower than that of the HFD group (Figure 3(c), HFD group *vs*. ISO-treated group). This result indicated that ISO could reduce the body weight of high-fat diet mice, which was a vital basis for ISO treatment of NAFLD.

**Figure 4:**
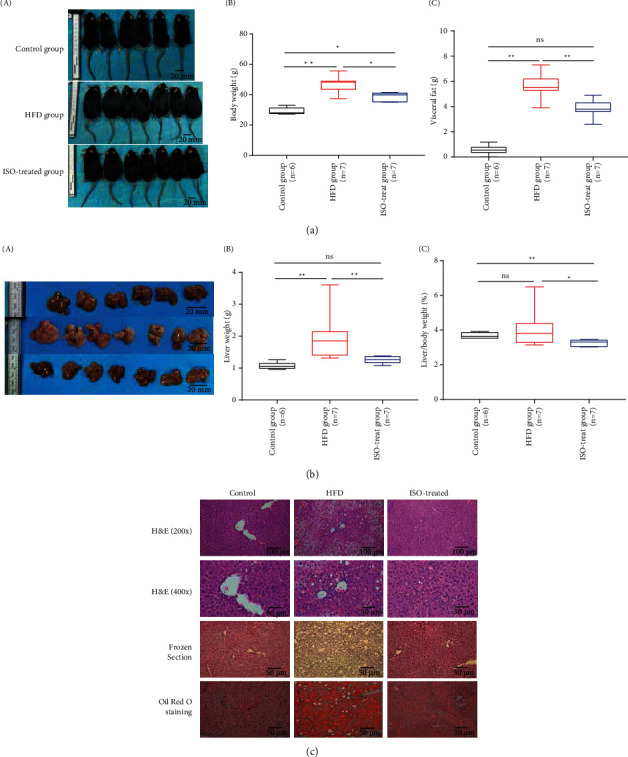
ISO treatment decreased liver steatosis in high-fat diet mice. (a) (i). The morphology pictures of mice. (a) (ii-iii). The body weight and visceral fat in three groups. (b) (i). The morphology pictures of liver. (b) (ii-iii). The liver weight and liver index (%) in three groups. The liver index means liver weight/body weight (%). (c) H&E, frozen section, and Oil Red O staining of liver tissue. The scale bar of H&E (400x) is 20 *μ*m. The scale bar of H&E (200x) is 100 *μ*m. The scale bar of frozen section and Oil Red O staining is 50 *μ*m.

After anesthesia, the mice were weighed and photographed. From the morphology of the mice, the HFD group was fatter than in the control group (Figure 4(a) i, control group *vs*. HFD group), and the results of body weight showed that the HFD group was significantly heavier than in the control group (Figure 4(a) ii, control group *vs*. HFD group). Particularly, ISO significantly reduced the body weight and body size of the high-fat diet mice compared with the model group (Figure 4(a) i, ii, HFD group *vs*. ISO-treated group). Meanwhile, we know that hepatic steatosis is closely associated with increased visceral fat [32]. After dissecting the mice, the visceral fat weight of the HFD group was significantly higher than that of the control group (Figure 4(a) iii, control group *vs*. HFD group), but ISO could significantly reduce the visceral fat weight of high-fat diet mice (Figure 4(a) iii, HFD group *vs*. ISO-treated group). According to the morphology of mice liver, the color of the livers in the HFD group was whiter than that in the control group (Figure 4(b) i, control group *vs*. HFD group), and the morphological changes in livers suggest that the high-fat diet caused liver steatosis in mice. The livers of the mice on the high-fat diet were significantly larger than those of the control group, suggesting that the high-fat diet not only increases body weight but also increases liver size. So, the body weight, liver weight, and visceral fat weight of mice in the high-fat diet group were significantly higher than those in the control group. On these indicators, liver steatosis was more severe in the high-fat diet group than in the control group. These indicators were reversed after the ISO-treated group compared with the HFD group (Figure 4(a) and 4(b), HFD group *vs*. ISO-treated group), suggesting that ISO treatment could reverse liver steatosis induced by a high-fat diet (Figure 4(b), HFD group *vs*. control group, control group *vs* ISO-treated group). We speculated that ISO treatment might reduce the liver index. In H&E staining, we observed that there were obvious ballooned lipid vacuoles in the liver tissues of the high-fat diet group (Figure 4(c), control group *vs* HFD group), indicating that there were lipid deposits in the liver tissues. The lipid deposits were significantly mitigated after ISO treatment (Figure 4(c), HFD group *vs*. ISO-treated group). This phenomenon was further confirmed by the Oil Red O staining and frozen section (Figure 4(c), control group *vs*. HFD group, HFD group *vs*. ISO-treated group). These liver histological changes demonstrated that ISO could significantly attenuate hepatic lipid accumulation induced by a high-fat diet and decrease the number of intracellular lipid droplets and hepatocyte balloons. These results suggested that ISO could improve body weight, liver weight, and fat accumulation in high-fat diet mice, and we suggested that ISO had a dramatic therapeutic effect on NAFLD.

### 3.4. ISO Improves Liver Function and Blood Glucose in High-Fat Diet Mice

**Figure 5:**
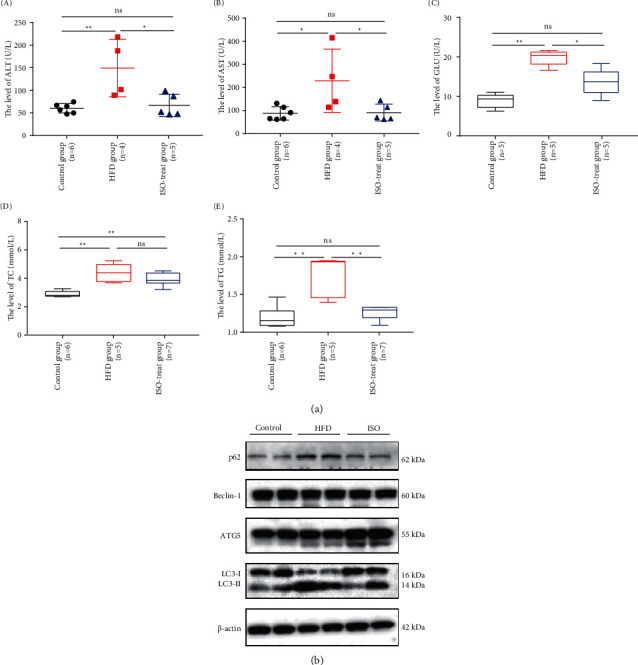
Blood indexes and the Western blot of liver. (a) Evaluation of liver function and blood glucose level markers after ISO treatment. (i–v). The level of ALT, AST, GLU, TC, and TG in mice. (b) ISO treatment activated autophagy to reverse liver steatosis in mice. The expression of autophagy-related proteins such as p62, beclin1, Atg5, LC3-II, and in mice liver analyzed by Western blot.

Studies have shown that liver steatosis will be accompanied by changes in blood indicators [33]. To explore the effects of ISO on the function of the liver and blood glucose in mice, we extracted serum from mouse blood and tested them with various blood index kits. We selected and examined ALT, AST, GLU, TC, and TG as a reference and tested the serum of mice (Figure 5(a)). The results of blood biochemical analysis showed that the levels of these indicators in the HFD group were significantly higher than those in the control group (Figure 5(a) i, ii, iii, iv, v, control group *vs*. HFD group), indicating that the high-fat diet led to liver function injury and raised blood glucose, but the blood biochemical indicators in the ISO-treated group were lower than those in the HFD group (Figure 5(a) i, ii, iii, iv, v, HFD group *vs* ISO-treated group). These experimental results suggested that ISO reversed the damage to liver function and the elevation of blood glucose caused by a high-fat diet. Therefore, we believed that ISO could improve liver function damage and insulin resistance caused by a high-fat diet, indicating that ISO had therapeutic potential on NAFLD.

### 3.5. ISO Attenuates the Inhibitory Effect of a High-Fat Diet on Autophagy

Through the above results, we observed that ISO improved liver steatosis caused by a high-fat diet, and we further learned that autophagy played a significant role in the treatment of NAFLD [9]. Next, we explored whether ISO could reduce liver steatosis in mice on a high-fat diet by activating autophagy. We extracted proteins from mice liver tissue and observed the changes in autophagy-related proteins. We examined the expression levels of the proteins using Western blot (Figure 5(b)), and the expression of p62 and LC3-II in the HFD group was significantly higher than in the control group (Figure 5(b), control group *vs*. HFD group). The expression level of p62 and LC3-II in the ISO-treated group was significantly lower than that in the HFD group (Figure 5(b), HFD group *vs*. ISO-treated group). ISO reduced the expression of p62 and LC3-II, suggesting that ISO improved autophagy inhibited by the high-fat diet *in vivo*. ISO might promote the degradation of autophagy-lysosome and thus reduce the accumulation of p62 and LC3-II protein. However, there was no significant difference in beclin-1 and ATG5 among the three groups (Figure 5(b), HFD group *vs*. control group, HFD group *vs*. ISO-treated group, control group *vs*. ISO-treated group), indicating that a high-fat diet and ISO treatment did not affect autophagosome formation.

## 4. Discussion

Recently, more and more people have paid attention to the treatment of NAFLD by Chinese herbal medicine [13]. Many drugs for the treatment of NAFLD were also extracted from Chinese herbal medicine, such as silymarin and berberine, which had been widely used to treat NAFLD [34, 35]. Many clinical studies also supported NAFLD treatment by Chinese herbal medicine. A growing number of studies supported the treatment of NAFLD in Chinese herbal medicine [36, 37]. The Chinese herbal medicine of flavonoids had anti-inflammatory and antioxidant stress effects, which provided a theoretical basis for treating NAFLD by flavonoids [17, 38]. ISO is a monomer of traditional Chinese medicine extracted from Acacia chinensis and is one of the active components of total flavonoid glycosides. There were few studies on the treatment of NAFLD by ISO. The main content of our study was that ISO reversed NAFLD by activating autophagy flux *in vivo* and *in vitro*.

At present, the pathogenesis of NAFLD is still largely unknown, and our *in vivo* and *in vitro* study provides an excellent theoretical basis. We found that ISO had a strong liver protective effect on NAFLD, and we considered that autophagy might be involved in the effect. Recently, we have seen that autophagy played an irreplaceable role in hepatic steatosis [39]. We conducted a series of experiments to verify this phenomenon.

We conducted a study with HepG2 cells. We found that PA-induced lipotoxicity played an essential role in the pathogenesis of NAFLD [25, 40]. We treated HepG2 cells with PA to establish the NAFLD lipid deposition cell model. It has been suggested that HepG2 cells can be used as a cell model for lipid deposition [27]. HepG2 cells treated with different concentrations of PA resulted in the accumulation of p62 and increase in LC3-II, but beclin-1 and ATG5 related to autophagosome formation did not change significantly, indicating that cell lipid deposition induced by PA did not affect autophagosome formation but inhibited the degradation of autophagy lysosomes, resulting in downstream damage of autophagy pathway. In short, ISO could reduce the accumulation of LC3-II and p62 after PA treatment, but ISO did not affect the expression of beclin-1 and ATG5.

We further verified the role of ISO in reducing hepatic steatosis and activating autophagy flux *in vivo*. We found that in mice ISO treatment significantly reduced hepatic steatosis, TG, TC, AST, ALT, and GLU levels. Compared with the HFD group, the liver lipid deposition was significantly reduced in the ISO-treated group, and Oil Red O staining showed a marked decrease in intracellular lipid droplets in liver tissue. These experiments suggest that ISO treatment could reverse the degree of hepatic adipose degeneration. Compared with the HFD group, the body weight, liver weight, liver index, and visceral fat weight of C57 mice in the ISO-treated group were decreased. These results suggest that ISO has a protective effect on NAFLD.

The autophagy of the liver in C57 mice fed with a high-fat diet was impaired [27]. The accumulation of p62 and the increase in LC3-II protein occurred simultaneously in the liver of C57 mice, indicating that the function of autophagy in C57 mice liver was impaired [41]. ISO treatment reduced the expression of p62 and LC3-II, which suggested that ISO could improve the inhibition of autophagy in the liver of mice. Through literature reviews, we found that the accumulation of p62 reflected the decrease in autophagy flux. Conversely, the activation of autophagy flux could decrease p62 expression [42]. LC3-II was also an autophagy substrate protein, and the increased expression of LC3-II could not simply be considered as autophagy activation or inhibition. It needed to evaluate the autophagy state together with p62 [43]. The accumulation of LC3-II might be interpreted as a result of inducing autophagy or blocking downstream of autophagy [43]. Therefore, in our study we considered that the decreased expression of p62 and LC3-II in ISO treatment might reflect the activation of autophagy flux.

According to literature studies, the mechanisms of increasing autophagy flux are as follows: firstly, promoting autophagosome formation; secondly, increasing the fusion of autophagosome and lysosome into autophagolysosome [43]. We found that ISO treatment reduced LC3-II and p62 levels but had no effect on the expression of beclin-1 and ATG5. Therefore, we believe that ISO is unlikely to activate autophagy by promoting upstream autophagosome formation. ISO may improve autophagy flux by increasing autophagosome-lysosome fusion, and ISO may play a role downstream of autophagy.

The activation of autophagy was helpful for the treatment of NAFLD. Autophagy activation could rapidly metabolize the lipid accumulation in liver cells, and autophagy played a vital role in liver lipid metabolism [44]. The imbalance of autophagy was closely related to the occurrence and development of liver diseases, and people had found that autophagy could also affect liver cirrhosis and even liver cancer [45]. Recent studies have shown that autophagy is closely associated with obesity and insulin resistance [46], which may also be a momentous factor in accelerating the progression of NAFLD after autophagy is affected.

In this experiment, it may be necessary to further observe the number of intracellular lipid droplets and autophagosomes in cells or liver tissues by scanning electron microscopy, to further explore the reversal of NAFLD through activation of autophagy by ISO. Finally, we summed it up. The results of *in vivo* and *in vitro* experiments showed that ISO might reverse hepatic steatosis through regulating the fusion of autophagosome and lysosome. The activation of autophagy can be used as a treatment for hepatic steatosis. These results suggest that ISO-mediated autophagy regulation may provide a novel strategy for the treatment of NAFLD.

## 5. Conclusion

In conclusion, our study showed that ISO reduced PA-induced intracellular lipid droplets in HepG2 cells *in vitro*, and ISO reversed liver steatosis induced by a high-fat diet *in vivo*, which might be related to ISO activation of the autophagy pathway. Our study suggested that ISO might be a potential treatment for NAFLD.

## Data Availability

The data used to support the results in this study are included within the article.

## Abbreviations

NAFLD: Nonalcoholic fatty liver disease
ISO: Isoschaftoside
PA: Palmitic acid
LC3B: Microtubule-associated protein 1 light-chain 3-beta
SQSTM1/p62: Sequestosome 1
ATG5: Autophagy-related protein 5
BSA: Bovine serum albumin
FBS: Fetal bovine serum
CQ: Chloroquine
SDS-PAGE: Sodium dodecyl sulfate polyacrylamide gel electrophoresis
PVDF: Polyvinylidene fluoride
ALT: Glutamic pyruvic transaminase
AST: Glutamic oxalacetic transaminase
GLU: Fasting blood glucose
TC: Total cholesterol
TG: Total triglycerides
P/S: Penicillin/streptomycin
U/L: Unit/litre
H&E: Hematoxylin and eosin.

## Supplementary Materials

Supplementary Materials In Supplementary Figure 1, the contents are the structural formula of isoschaftoside and the quantitative analysis of Figure 1(c). In Supplementary Figure 2, the contents are immunofluorescence for LC3B and the enzyme activity of apoptosis-related gene caspase 3.
