# Supplementary material for: Isoschaftoside Reverses Nonalcoholic Fatty Liver Disease via Activating Autophagy In Vivo and In Vitro
Source: Evid Based Complement Alternat Med. 2022 Jun 27;2022:2122563. doi: 10.1155/2022/2122563 (PMC9252632; doi:10.1155/2022/2122563)
Supplement: Supplementary Materials — In Supplementary Figure 1, the contents are the structural formula of isoschaftoside and the quantitative analysis of Figure 1(c). In Supplementary Figure 2, the contents are immunofluorescence for LC3B and the enzyme activity of apoptosis-related gene caspase 3. [file 2122563.f1.zip › 2122563.f1/Supplementary Figure .docx]

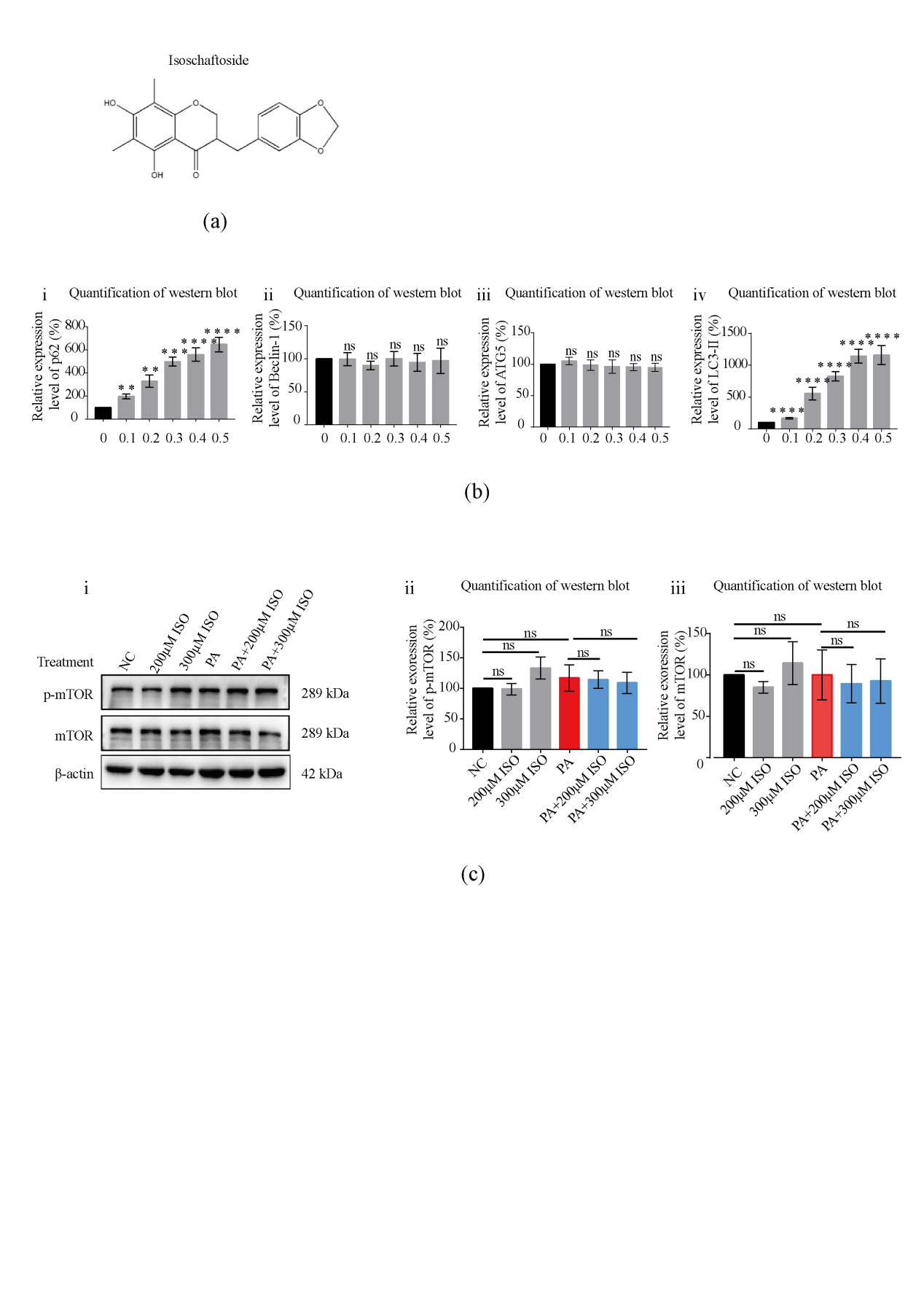


Supplementary FIGURE 1: (a) The structural formula of Isoschaftoside (C26H28O14). (b) Western blot was used to verify the effect of PA on autophagy-related proteins and quantitative analysis of FIGURE 1C. (c) Effects of PA and ISO on mTOR and p-mTOR, and quantification of western blot.


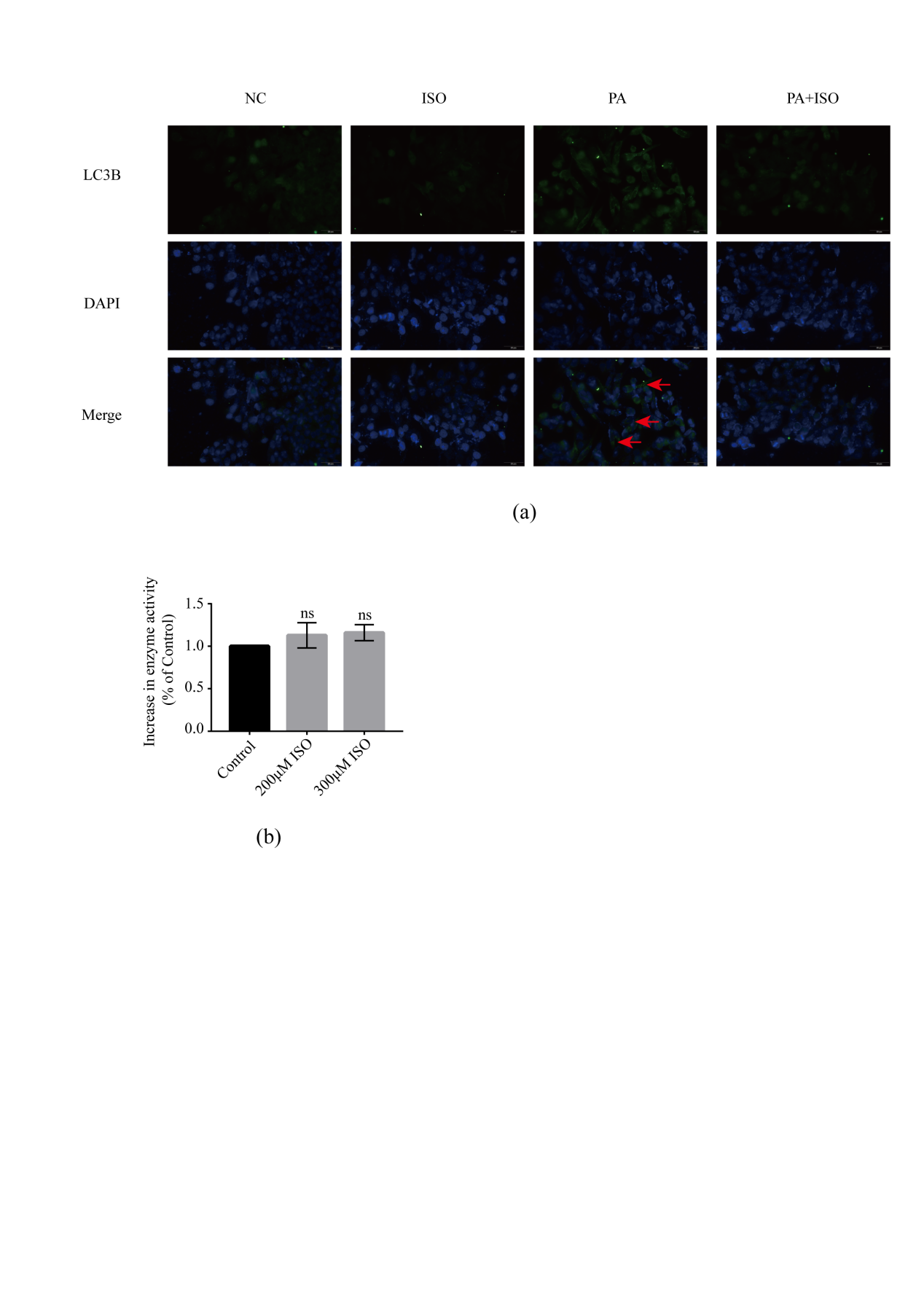


Supplementary FIGURE 2: (a) Imunofloursence for LC3B : Green fluorescent protein represents the accumulation of LC3B protein in the cell, marked in red. (b) Effects of ISO on the enzyme activity of apoptosis related gene Caspase-3.
