# Supplementary figures and images for: Isoschaftoside Reverses Nonalcoholic Fatty Liver Disease via Activating Autophagy In Vivo and In Vitro
Source: Evid Based Complement Alternat Med. 2022 Jun 27;2022:2122563. doi: 10.1155/2022/2122563 (PMC9252632; doi:10.1155/2022/2122563)

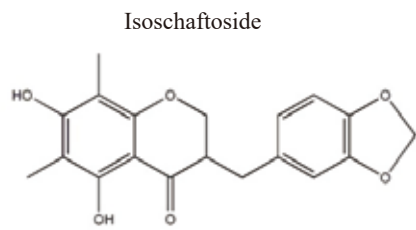

(a)

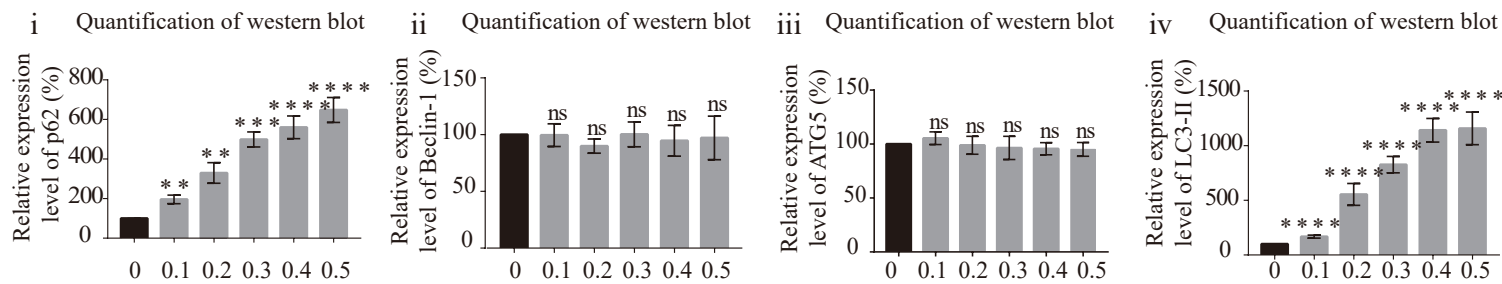

(b)

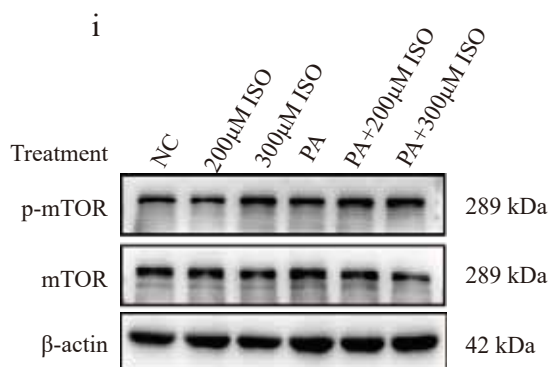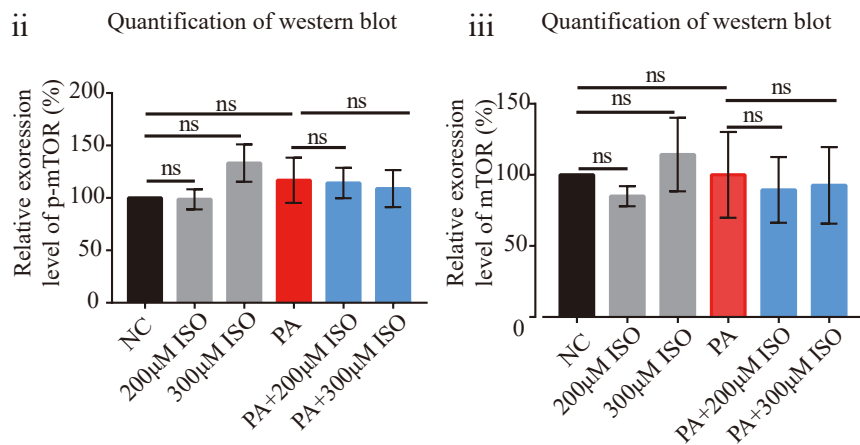

(c)

Supplement: Supplementary Materials — In Supplementary Figure 1, the contents are the structural formula of isoschaftoside and the quantitative analysis of Figure 1(c). In Supplementary Figure 2, the contents are immunofluorescence for LC3B and the enzyme activity of apoptosis-related gene caspase 3. [file 2122563.f1.zip › 2122563.f1/supplementary-1.pdf]

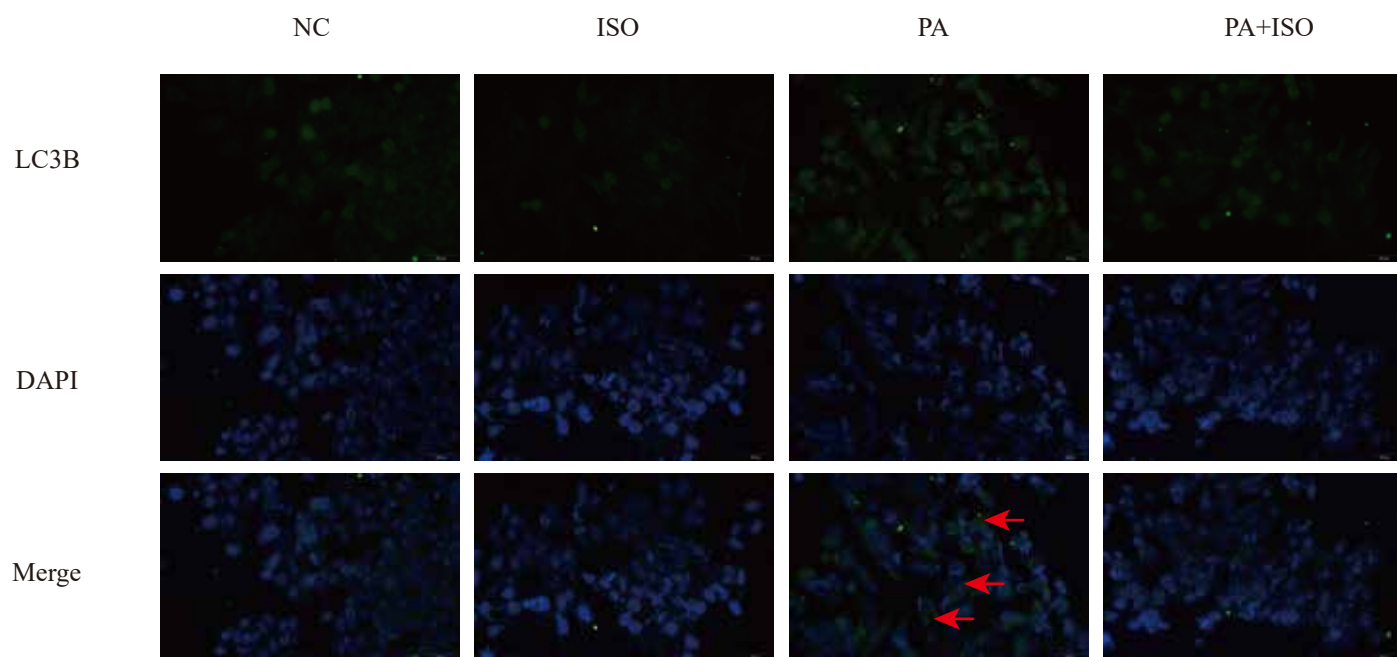

(a)

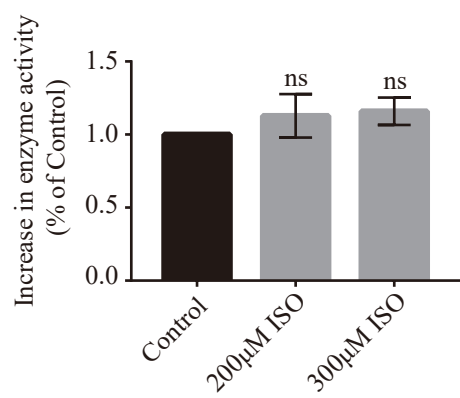

(b)

Supplement: Supplementary Materials — In Supplementary Figure 1, the contents are the structural formula of isoschaftoside and the quantitative analysis of Figure 1(c). In Supplementary Figure 2, the contents are immunofluorescence for LC3B and the enzyme activity of apoptosis-related gene caspase 3. [file 2122563.f1.zip › 2122563.f1/supplementary-2.pdf]
